# Treatment seeking behaviours, antibiotic use and relationships to multi-drug resistance: A study of urinary tract infection patients in Kenya, Tanzania and Uganda

**DOI:** 10.1371/journal.pgph.0002709

**Published:** 2024-02-16

**Authors:** Keina Sado, Katherine Keenan, Areti Manataki, Mike Kesby, Martha F. Mushi, Stephen E. Mshana, Joseph R. Mwanga, Stella Neema, Benon Asiimwe, Joel Bazira, John Kiiru, Dominique L. Green, Xuejia Ke, Antonio Maldonado-Barragán, Mary Abed Al Ahad, Kathryn J. Fredricks, Stephen H. Gillespie, Wilber Sabiiti, Blandina T. Mmbaga, Gibson Kibiki, David Aanensen, V. Anne Smith, Alison Sandeman, Derek J. Sloan, Matthew T. G. Holden, on behalf of HATUA Consortium

**Affiliations:** 1 University of St Andrews, St Andrews, United Kingdom; 2 Catholic University of Health and Allied Sciences, Mwanza, Tanzania; 3 Makerere University, Kampala, Uganda; 4 Mbarara University of Science and Technology, Mbarara, Uganda; 5 Kenya Medical Research Institute, Nairobi, Kenya; 6 Kilimanjaro Clinical Research Institute, Kilimanjaro Christian Medical Centre, Moshi, Tanzania; 7 Kilimanjaro Christian Medical University College, Moshi, Tanzania; 8 Africa Excellence Research Fund, London, United Kingdom; 9 Oxford Big Data Institute, Oxford, United Kingdom; University of Ottawa Faculty of Medicine, CANADA

## Abstract

Antibacterial resistance (ABR) is a major public health threat. An important accelerating factor is treatment-seeking behaviour, including inappropriate antibiotic (AB) use. In many low- and middle-income countries (LMICs) this includes taking ABs with and without prescription sourced from various providers, including health facilities and community drug sellers. However, investigations of complex treatment-seeking, AB use and drug resistance in LMICs are scarce. The Holistic Approach to Unravel Antibacterial Resistance in East Africa (HATUA) Consortium collected questionnaire and microbiological data from adult outpatients with urinary tract infection (UTI)-like symptoms presenting at healthcare facilities in Kenya, Tanzania and Uganda. Using data from 6,388 patients, we analysed patterns of self-reported treatment seeking behaviours (‘patient pathways’) using process mining and single-channel sequence analysis. Among those with microbiologically confirmed UTI (n = 1,946), we used logistic regression to assess the relationship between treatment seeking behaviour, AB use, and the likelihood of having a multi-drug resistant (MDR) UTI. The most common treatment pathway for UTI-like symptoms in this sample involved attending health facilities, rather than other providers like drug sellers. Patients from sites in Tanzania and Uganda, where over 50% of patients had an MDR UTI, were more likely to report treatment failures, and have repeat visits to providers than those from Kenyan sites, where MDR UTI proportions were lower (33%). There was no strong or consistent relationship between individual AB use and likelihood of MDR UTI, after accounting for country context. The results highlight the hurdles East African patients face in accessing effective UTI care. These challenges are exacerbated by high rates of MDR UTI, suggesting a vicious cycle of failed treatment attempts and sustained selection for drug resistance. Whilst individual AB use may contribute to the risk of MDR UTI, our data show that factors related to context are stronger drivers of variations in ABR.

## Introduction

Antibacterial resistance (ABR) is a major global health challenge which compromises our ability to treat infections with antibiotics (ABs) [1]. ABR was associated with an estimated 5 million deaths globally in 2019, and the annual mortality rate from ABR is expected to increase further by 2050 [2,3]. The burden is greatest in low- and middle-income countries (LMICs) [2], which experience a higher prevalence of infectious diseases, and have fewer technical and financial resources to deal with multi-drug resistant (MDR) pathogens. Many studies have identified increased AB use, and prior AB use, as risk factors for ABR infections, driving resistance through exerting bacterial selection pressures within individuals and communities [4–7]. Deeper understanding of the dynamics of treatment-seeking, AB use and ABR infections in LMIC settings is therefore crucial to tackle the problem. This study focuses on urinary tract infections (UTIs) as a lens for understanding treatment-seeking and ABR more generally. We concentrate on UTIs because they are common bacterial illnesses which are usually treated with ABs, and misuse of ABs (and more specifically, empiric AB use to treat UTIs) leads to ABR of the uropathogens responsible (typically *Escherichia coli* and other *Enterobacteriacae*). Increasing rates of ABR in uropathogens is a growing global challenge.

Healthcare and treatment landscapes in LMICs are sometimes described as ‘pluralistic’ [8], which typically means there are multiple sources of public and private clinics, alongside pharmacists, drug sellers, and complementary and traditional/herbal medicine providers. This multi-layered context of care provision overlaps with models of medical syncretism, which describe how patients make treatment decisions drawing on hybrid logics [9], composed of both biomedical understandings and local knowledge/beliefs present in different contexts [9,10]. Health decision-making in many LMICs is further influenced by a number of individual and structural factors, including under-resourced public health provision, stock-outs of medicines and multi-layered systems of healthcare insurance [10,11]. This potentially leads to great diversity in pathways to AB use among patients, something which has been recently highlighted as a research gap [8].

Existing research suggests that AB self-medication from drug shops is very common in parts of Asia and Sub-Saharan Africa [12–16]. This is facilitated, in some areas, by the ease of purchasing ABs without prescription at community pharmacies and drug shops [17], and the possibility of buying partial courses which can result in sub-optimal treatment dosing. Studies also suggest that consumption of leftover ABs (i.e. previously obtained but unconsumed) is common [15]. In East Africa, high community prevalence of UTIs, combined with AB self-medication, may further exacerbate ABR [17], particularly as self-management for UTI symptoms is reportedly very common [14,15]. Thus far, there have been no studies investigating the interrelationship between patient AB use and ABR in East Africa, a region with high levels of ABR [2,18], high prevalence of non-prescription drug sales [19] and complex, hybrid treatment landscapes.

In previous studies, AB use or ‘misuse’ has often been measured simply as, for example, a binary indicator of any type of self-medication [14]. However, situating treatment-seeking behaviours in their appropriate social and temporal context is vital. AB self-medication may occur as part of a longer sequence of treatment attempts [20], or in parallel to other types of AB use and health treatments, emphasising the importance of a longitudinal, pathway based approach [21]. Pathway analysis has been used to investigate care for tuberculosis [22], abortion [23], and cancer [24] but has rarely been used to understand AB use [13]. Untangling cause and effect between high AB use and ABR is challenging, especially where ABR infections are more common, and repeat AB use may be a reaction to treatment failure. Appropriate data and methods are needed to capture the behavioural nuance inherent in such patient pathways. This study takes advantage of exceptionally detailed linked quantitative social and microbiological data collected as part of a multi-country interdisciplinary consortium “Holistic Approach to Unravel Antibacterial Resistance in East Africa (HATUA)” which aimed to delineate the drivers of antibiotic resistance in this community [25].

In this study, we address two research questions: 1) What are the main characteristics of treatment-seeking pathways among patients with UTI-like symptoms in Kenya, Tanzania, and Uganda? and 2) How do treatment-seeking behaviours and individual AB use relate to the likelihood of developing an UTI caused by a multi-drug resistant (MDR) pathogen (i.e. resistant to at least 3 classes of antibiotic)?

## Materials and methods

### Data and sample

**Fig 1:**
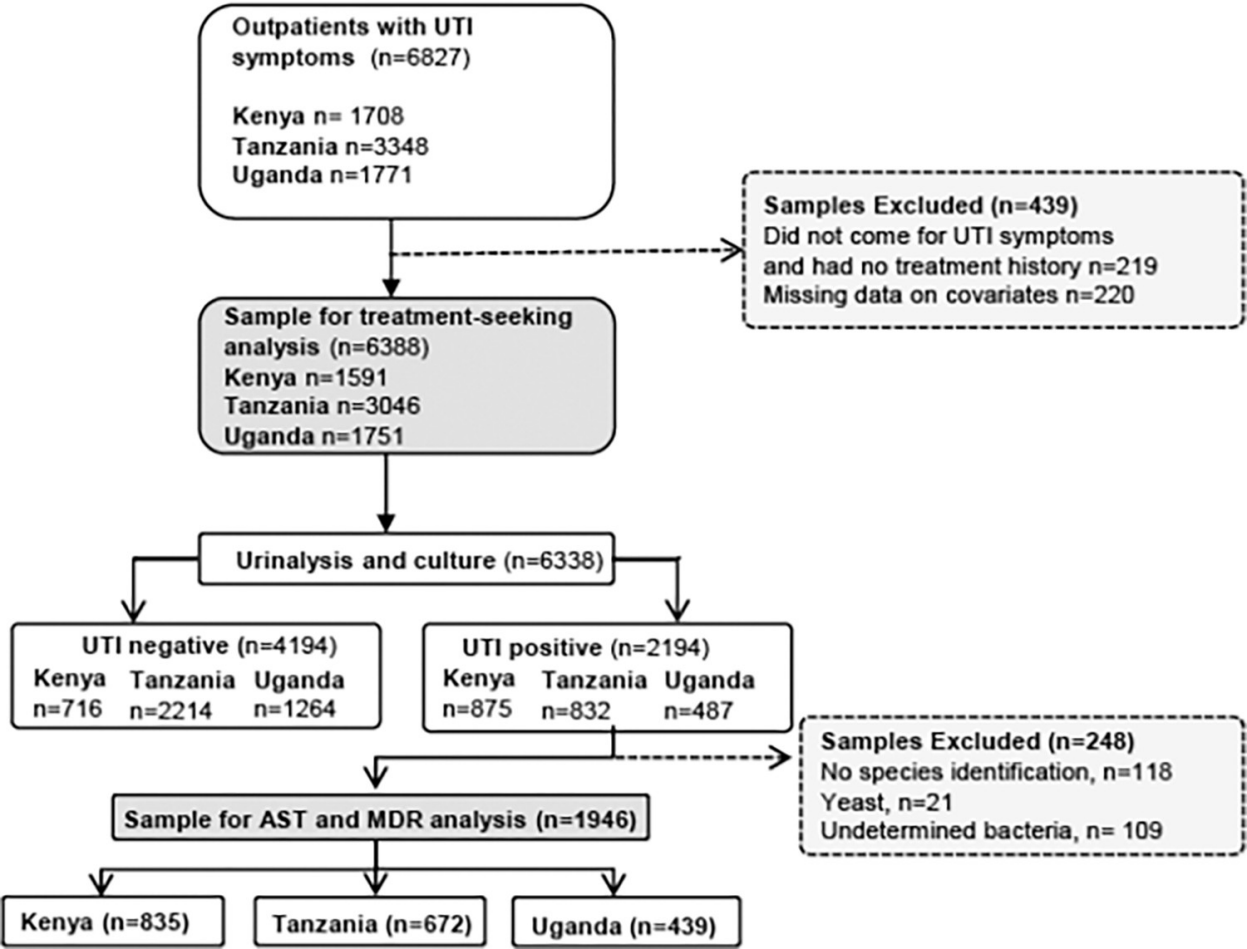
Selection of the analysis samples.

The original sample consisted of 6,827 adult outpatients presenting to the Doctor with UTI-like symptoms, aged 18 years and over, and pregnant adolescents aged 14–17 years (who comprised 1% of the sample), who were recruited from three sites in Kenya (Makueni, Nairobi, and Nanyuki), Tanzania (Kilimanjaro, Mbeya, and Mwanza), and Uganda (Mbarara, Nakapiripirit, and Nakasongola), between February 2019 and September 2020. Doctors identified adult patients for recruitment based on self-reported symptoms, which included lower abdominal or flank pain, fever, combined with urinary symptoms (burning when urinating, frequent urination, urgency); hematuria or pyuria was assessed using dipstick testing. We recruited patients at medical facilities that were predominantly government-funded (see S1 Table for a full explanation). In all sites, less than 1% of those identified for inclusion declined to participate. Ethical approval was obtained from National and Institutional Research Ethics Committees (see protocol [25]). We collected quantitative questionnaire data and recorded laboratory data using EpiCollect 5 [26], full details of which are in the protocol [25]. Questionnaire and microbiological data were linked using anonymous patient IDs. The process of sample selection is shown in Fig 1. For the analysis of drug resistance, we selected only those with microbiologically confirmed UTI and data on drug resistance.

### Microbiological identification of UTI and antibiotic sensitivity testing (AST)

Full details of microbiological methodology, standardised protocols, and quality assurance is available in another paper [17]. The same paper also provides a detailed overview of microbiological results for all patients (including children and outpatients). For this study, which only includes adult outpatients, they were asked to provide a clean catch mid-stream urine sample, which underwent microbiological culture, pathogen identification and AST. Urinary tract infection positive samples (UTI+) were defined by the presence of >10^4^ colony-forming units per millilitre (CFU/mL) of one or two uropathogens. This was chosen after consultation with East African microbiologists to be consistent with diagnostic cutoffs used in the sites, thereby providing clinically relevant results. For UTI positive patients, susceptibility to the tested antibiotics (see S2 Table for a full list) was done by the disk diffusion method, and determined using breakpoints (zone diameter interpretive criteria) indicated in the 2021 CLSI guidelines (M02 document) [27]. Multidrug-resistant (MDR) bacteria were defined as urinary isolates resistant to at least one agent in three or more categories of antimicrobial agents, following the European Centre for Disease Prevention and Control (ECDC) guidelines [28] with some modifications. Specifically, nitrofurantoin and trimethoprim, two antibiotics routinely used for treating UTIs that are not included in the ECDC guidelines, were also considered for estimating MDR. In addition, for those species/genera not incorporated in the ECDC guidelines, i.e. *Salmonella*, *Shigella* and *Streptococcus*, the MDR rates were calculated as above, but considering the resistance to a selected pool of tested antibiotics (S2 Table). Isolates that showed intermediate resistance to a given antibiotic were considered resistant. AB susceptibility/MDR rates were calculated in R version 4.1.1 [29].

### Variables: Self-reported treatment-seeking behaviours

**Fig 2:**
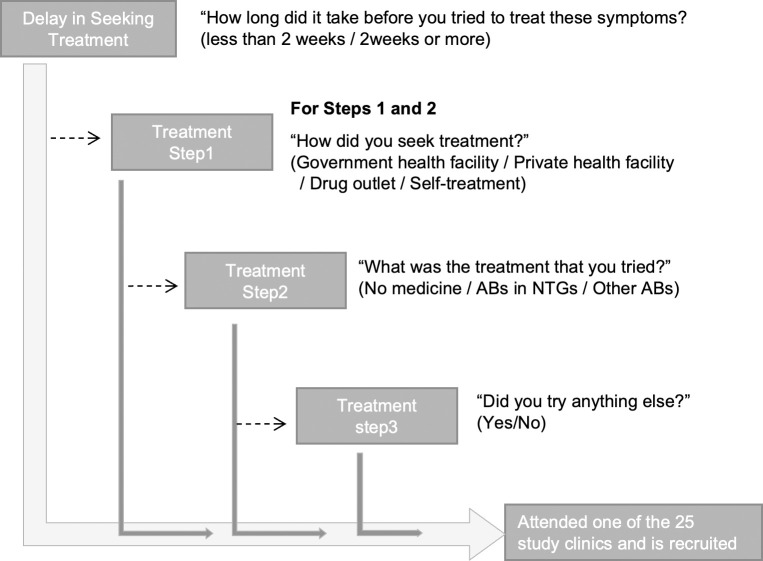
Treatment-seeking questions from the HATUA questionnaire and derived outcomes in this study. **Footnote:** Adapted from another publication [11], published by the same authors. We retain the copyright and authorise this use.

Patients answered a questionnaire on treatment-seeking for UTI symptoms, AB use practices and attitudes, and sociodemographic characteristics. Fig 2 illustrates the structured questionnaire used to collect treatment-seeking data for the UTI symptoms, which identified the types of providers consulted, treatments/ABs taken, and reasons for these choices prior to attending the recruitment clinic. We derived a variable for the number of steps in the pathway (1 step, i.e. came straight to recruitment clinic, 2 steps, i.e. took one additional step before coming to recruitment clinic, and 3 or more steps i.e. took 2 or more steps before being recruited). During the interview, patients self-reported the names of medicines they had taken to treat their UTI-like symptoms, and were also prompted using a drug bag or drug card developed specifically for each site [30]. Subsequently, we assessed whether the AB they reported was recommended for treating UTI in adult outpatients according to National Treatment Guidelines (NTGs) for their country (see S3 Table).

### Other variables

We included patients’ self-reported sex (male/female), age (categorised into <25; 25–34; 35–44; 45–54; 55–64; 65+ years), and education level (none, primary, secondary, and tertiary). We also included a binary variable measuring use of any other ABs (apart from those reportedly taken to treat UTI symptoms) in the previous 6 months.

### Ethics approval and consent to participate

The study received ethical approval from the University of St Andrews, UK (number MD14548, 10/09/19); National Institute for Medical Research, Tanzania (number 2831, updated 26/07/19); CUHAS/BMC Research Ethics and Review Committee (number CREC /266/2018, updated on 02/2019); Mbeya Medical Research and Ethics Committee (number SZEC-2439/R.A/V.1/303030); Kilimanjaro Christian Medical College, Tanzania (number 2293, updated 14/08/19); Uganda National Council for Science and Technology (number HS2406, 18/06/18); Makerere University, Uganda (number 514, 25/04/18); and Kenya Medical Research Institute (04/06/19, Scientific and Ethics Review Committee (SERU) number KEMRI/SERU/CMR/P00112/3865 V.1.2). For Uganda, administrative letters of support were obtained from the district health officers to allow the research to be conducted in the respective hospitals and health centres. All participants provided written informed consent to participate.

### Analytical approach

#### Characterising treatment seeking behaviours

We employed computational methods of process mining and sequence analysis that are designed to detect patterns in process /flow data. The application of these methods to clinical healthcare data in LMICs is novel. Process mining is typically used to analyse event sequences automatically recorded in high-income settings, such as electronic inpatient records [27]. Sequence analysis is more commonly applied in biological genetics and sociology [31], but its use to analyse healthcare processes is being developed [32].

#### Process mining

**Fig 3:**
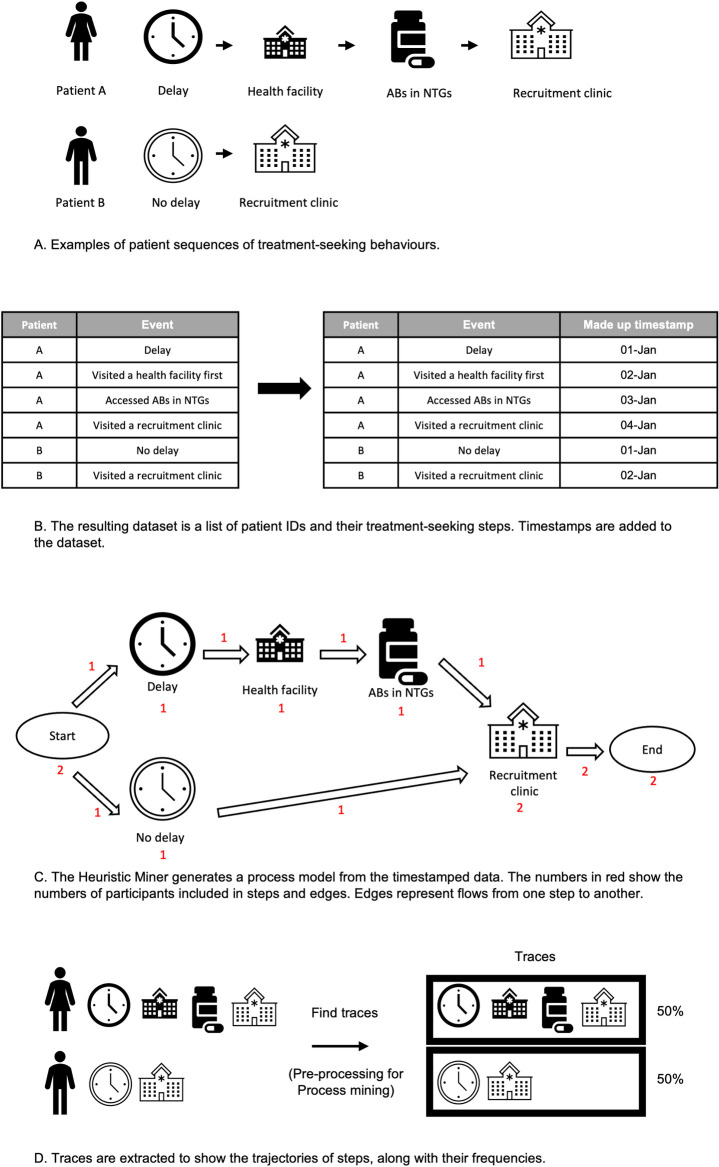
A-D: Stages in data management, analysis and visualization for process mining. Attributions for the open-source images can be found inS4 Table.

Self-reported responses shown in Fig 2 were organised in a temporal sequence for each patient, capturing delay (or not)-> step 1->treatment at step 1-> step 2 ->treatment at step 2->step 3. Patients who arrived at clinic at an earlier stage had shorter sequences. We assigned timestamps that reflected temporal sequences of individual patients. We also merged some categories of variables due to low frequency. For example, for the question “How did you seek treatment?”, we merged responses “Visiting government health facility” and “Visiting private health facility” into “Visiting health facility”. We employed the Heuristic Miner using the PM4Py package in Python [33]. The process is visually described in Fig 3. The output of the heuristic miner is a process model that captures the control-flow relations between tasks that are observed in the data, including the number of patients that moved from one step to another. In our study, we transformed the process model into a Sankey diagram that provides a general overview of all treatment seeking pathways. We visualised the most common pathways by analysing traces (i.e. sequences of steps) with the use of ProM [34] software. We compared the numbers/proportions of patients taking common trajectories and qualitatively compared the traces across countries.

#### Sequence analysis

Next, we used single-channel sequence analysis (WeightedCluster library in R [35]) to cluster patients with similar treatment-seeking pathways. To derive the clusters, we used the variables shown in Fig 2, subsequently recoded, as follows: (delay (yes/no), types of provider at step 1 and 2 (health facility/drug outlet/self-treatment), treatment at step 1 and 2 (ABs from NTGs/ other ABs/ no ABs), and number of steps (1,2,3+). In the preliminary stages of analysis, we noticed that the clustering was strongly driven by the number of steps. Therefore, to allow us to investigate other aspects of treatment seeking, we repeated the clustering exercise separately among those who took 1 step, 2 steps, and 3 or more steps. Following others [36], we used the optimal matching algorithm to calculate dissimilarity measures. Because all sequences in each subgroup have the same number of steps, costs for insertion/deletion were not required. Substitution costs were set to a constant value (the default). The optimal number of clusters were evaluated using two cluster quality measures: Point Biserial Correlation (PBC), and Weighted Average Silhouette width (ASWw) [35]. We assessed the characteristics of cluster membership by conducting a bivariate analysis assessing the associations between age, gender, education, country and cluster membership, using chi square tests for difference.

#### Associations between social factors, behaviours and MDR status

To investigate associations between treatment seeking and MDR, we used a subset of UTI positive patients with valid, linked data on MDR, treatment-seeking and socio-demographic characteristics (n = 1,946). We first cross-tabulated separate facets of treatment-seeking characteristics (delay, types of provider at every step, treatments at every step, number of steps) and socio-demographics (age, gender, education, country) with MDR status, and conducted chi-square tests for difference. Then, we assessed the association between cluster membership (derived from the sequence analysis described above) and MDR status using multivariable logistic regression, stratified by country.

## Results

### Sample description

To analyse patterns in UTI-treatment-seeking pathways, we excluded 219 patients who came to the recruitment clinic for non-UTI symptoms and had not attempted to treat their symptoms, leaving n = 6,608 patients. After further exclusions for missing covariate data (n = 220), the remaining 6,388 patients were composed of 1,591 (24.9%), 3,046 (47.7%), 1,751 (27.4%) from Kenya, Tanzania and Uganda respectively (see Fig 1). S5 Table shows the characteristics of patients included in the two different analysis samples (stratified by country in S6–S8 Tables). Considering the sample used for pathway analysis (n = 6,338), nearly half of the patients were recruited in Tanzania (47.7%), most were female, and of reproductive age (<44 years). Overall, most patients had either primary or secondary level education, with much smaller proportions with no or higher education. In terms of treatment seeking pathways, while many patients (45.1%) had come straight to the recruitment clinic, the majority (54.9%) had taken at least one additional treatment step prior to recruitment into the HATUA study. Approximately 30% of patients had already taken ABs to treat their UTI-like symptoms, and over 60% reported having taken ABs in the previous 6 months. Of the total patients, 34.3% of participants had a UTI pathogen microbiologically confirmed, and of these, nearly half (47.9%) had an MDR pathogen responsible for their infection.

To analyse associations with MDR (shaded green box in Fig 1), we further restricted the sample to microbiologically confirmed UTI patients with AST data for the pathogen identified. We excluded patients if the pathogen or causative bacteria was unknown, or where a breakpoint for disk diffusion test was unavailable. This resulted in a sample of n = 1946 which had slightly different distributions to the sample of all patients (S4–S7 Tables). For example, a higher proportion came from Kenya and fewer from Tanzania, and women and older people made up a larger fraction of the sample. This is related to the higher rates of confirmed UTI in those different subgroups influencing their eventual inclusion into the sample with MDR data.

### Characteristics of treatment-seeking pathways

**Fig 4:**
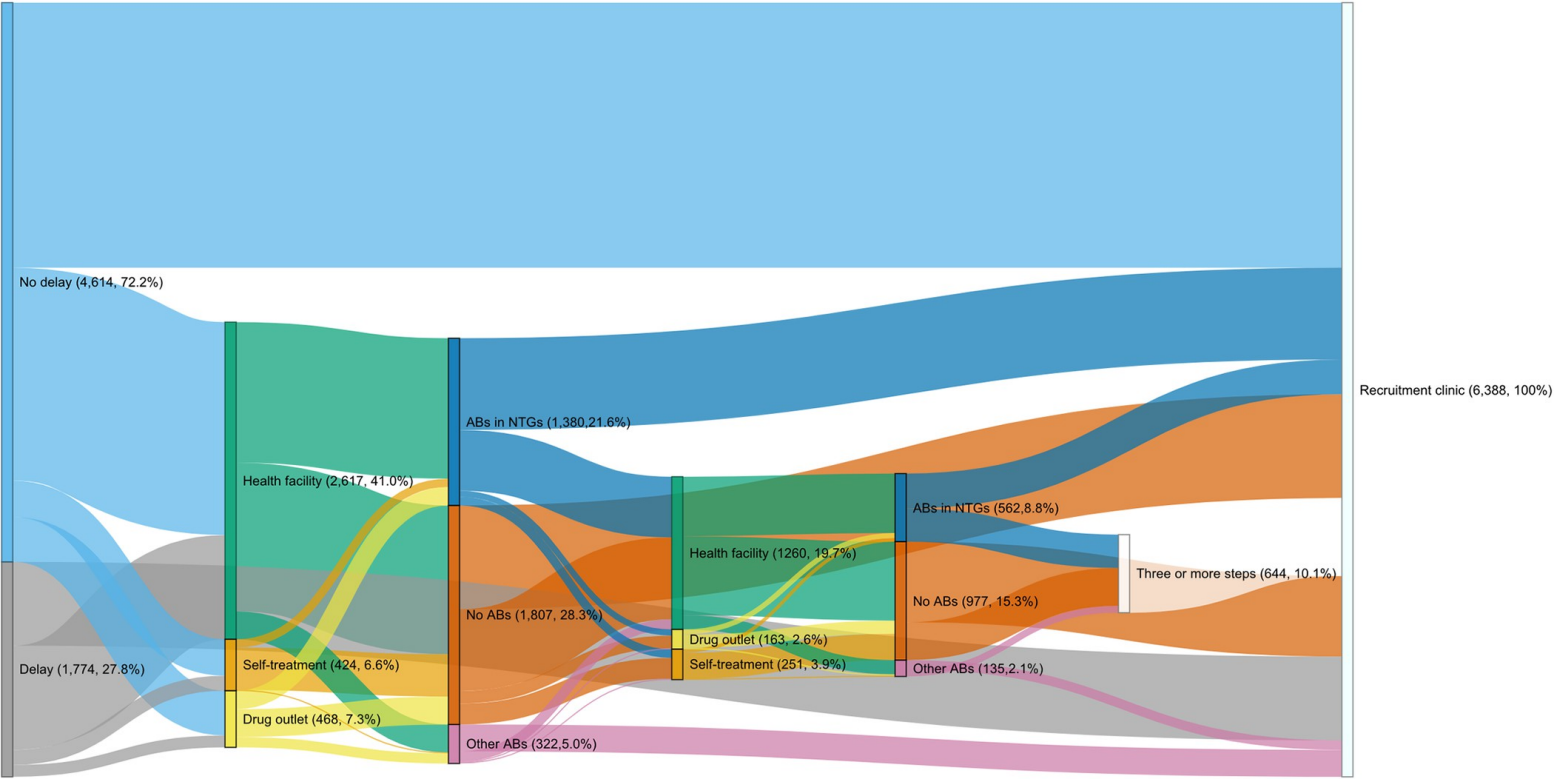
Treatment seeking pathways visualized in a Sankey plot among patients in Kenya, Tanzania and Uganda. The numbers by each node represent the absolute number and percentage of participants who took that step. The width of the arcs represents the frequency of the flow.

Fig 4 visualizes the treatment-seeking pathways derived from process mining for all countries combined. The majority (72%) of our patients accessed care within 2 weeks of recognizing their symptoms, while 28% waited longer than 2 weeks. The most common first step was visiting one of the study recruitment clinics (46% of patients), and the second most common was visiting other health facilities (41%), followed by visiting drug outlets (7%) and self-treatment (7%). At every step, attending health facilities, either the recruitment clinic or another government-funded facility, were the predominant steps (it is important to emphasize that our clinic-based recruitment strategy has likely led to bias in this direction). Finally, 644 (10% of all participants) tried three or more things before visiting the recruitment clinic. Among patients who did something before visiting the recruitment clinic (3,509 patients), at the first step, 39% took ABs recommended in the NTGs for UTI, 9% took other ABs, and 52% took other medications or nothing at all. At the second step (n = 1,674), there were similar proportions (33% took ABs in NTGs, 8% took other ABs).

Using the Trace Explorer function, we detected 183 unique treatment seeking patterns. Logically, shorter pathways were the most common because there was less opportunity for variation. The most frequently taken pathways were visiting the recruitment clinic either within 2 weeks of noticing symptoms (34% of patients), or after 2 weeks (11%). Visiting other types of clinics (government or private) and taking no medication or ABs in NTGs represented the third, fourth and fifth most-common pathways. It was only at the sixth most common when non-healthcare facility based treatments emerged. The majority of treatments taken were either ABs in NTGs, or other medicines (taking other ABs was the least common type of treatment), and these were mainly sourced after visiting healthcare facilities.

**Fig 5:**
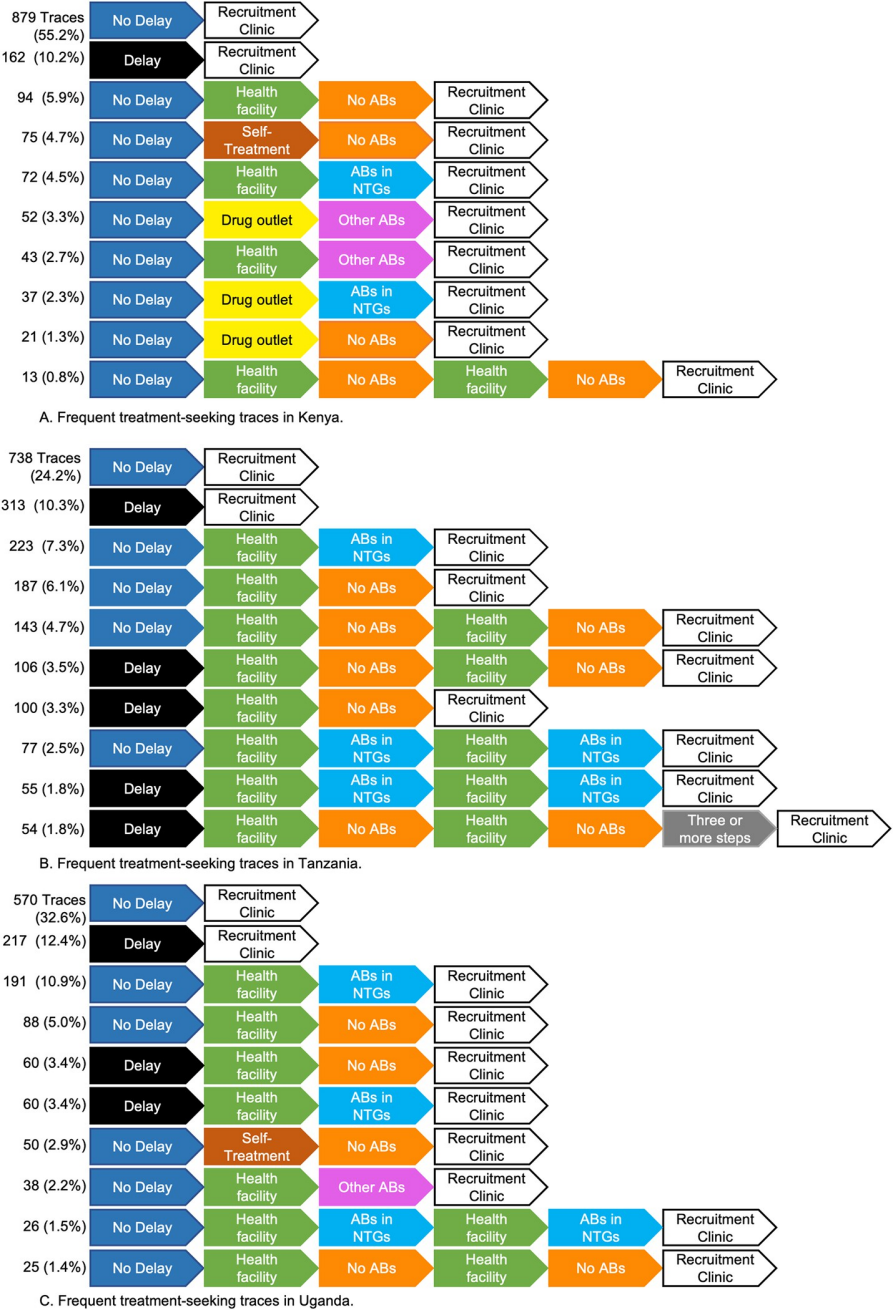
Ten most common treatment-seeking patterns visualized in Kenya (panel A), Tanzania (panel B) and Uganda (panel C). Number on the left represents the number of participants who experienced the trace. The percentage shows the proportion of participants who experienced the trace compared to the total patients from that country.

We considered country-level differences in the 10 most common pathways (see Fig 5) and this showed that Kenyan patients were more likely to access care without delay and directly at the recruitment clinic than those in Tanzania or Uganda (55% of Kenyan patients compared with 24% in Tanzania and 33% in Uganda). On the other hand, Kenyan patients who did not go straight to recruitment clinic were the most likely to try self-treatment (5%) or visit a drug outlet (4%) first. Among Tanzanian patients, the most common trajectories all featured healthcare facilities visits, but patients were more likely to have 2 and 3 step pathways than other countries (>10% of all sequences). Ugandan patients had simpler and more healthcare-dependent pathways than in Tanzania.

**Fig 6:**
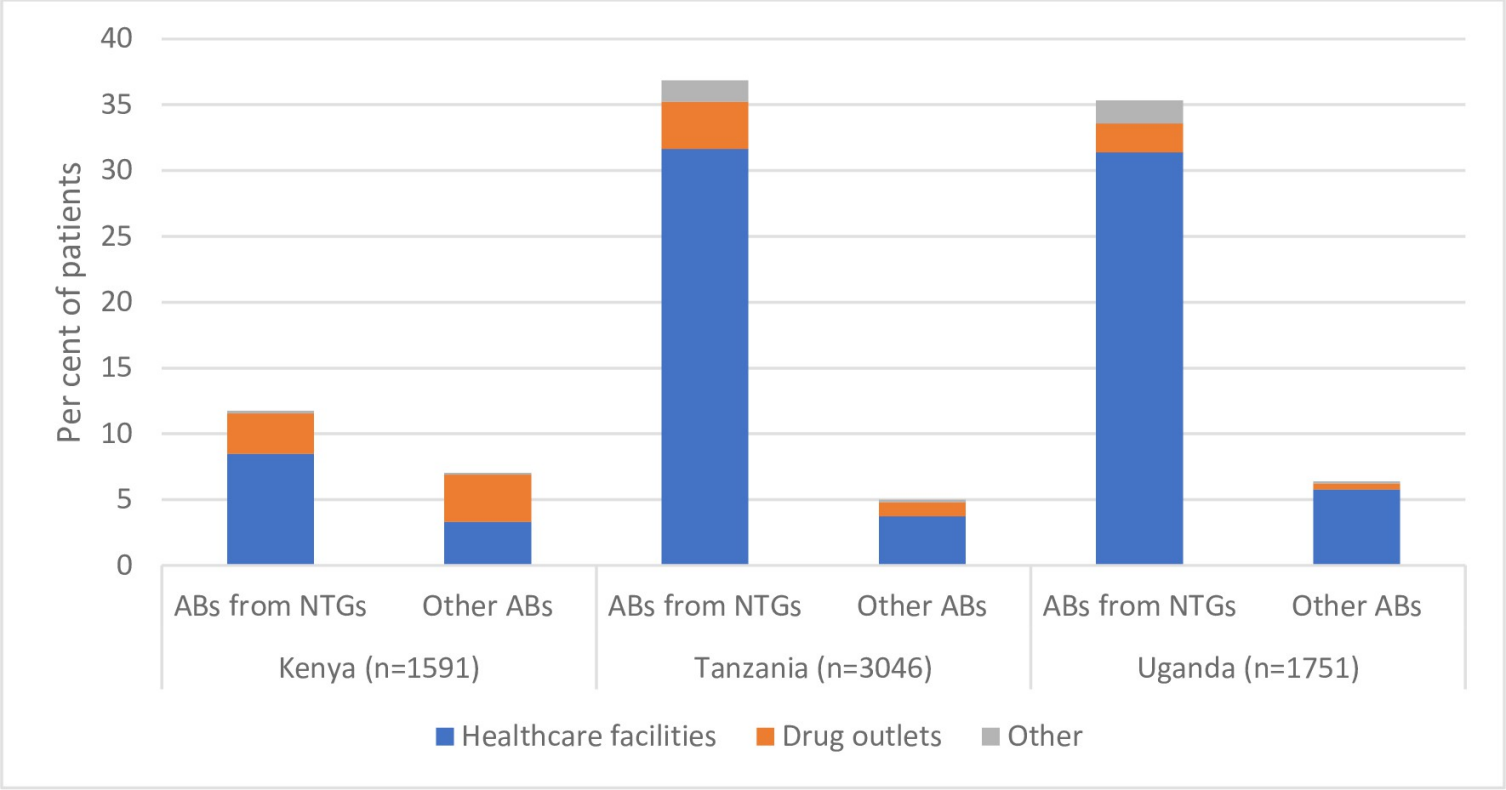
Percentage of patients taking ABs for their UTI-like symptoms at any point prior to being recruited, by country and type of provider.

Fig 6 shows the total distribution of patients taking any AB courses during the pathway, whether they were in the NTGs for UTI or not, and whether they were taken after a visit to healthcare facilities, drug outlets, or other. Overall, taking any type of ABs was less likely to occur in Kenya than in Tanzania or Uganda (a finding driven by the generally shorter pathways Kenyan patients reported). However, Kenyan patients had the highest rate of taking ABs not in the NTGs, and were more likely to access these from drug outlets, than in the other countries. In all countries, taking ABs was most likely to happen after a visit to a healthcare facility. Kenya patients were proportionally more likely to visit pharmacies/drug shops prior to taking ABs, than in Tanzania or Uganda. Patients with no microbiologically confirmed UTI were just as likely to have taken ABs to treat their UTI-like symptoms as those with a UTI confirmed (see Table 1). Of all the patients who took ABs, two thirds (66%) were patients with no confirmed UTI.

**Table 1:** AB use and UTI status, across 3 countries.

	Kenya		Tanzania		Uganda		TOTAL	
	UTI status		UTI status		UTI status		UTI status	
	Neg row %	Pos row%	Neg row %	Pos row%	Neg row %	Pos row%	Neg row %	Pos row%
**ABs from NTGs**	52.3	47.8	72.8	27.2	72.2	27.8	70.1	29.9
**Other ABs**	39.1	60.9	74.0	26.0	71.4	28.6	63.9	36.1
**No ABs**	44.5	55.5	72.5	27.5	72.3	27.8	64.4	35.7
**TOTAL**	45	55	72.7	27.3	72.2	27.8	65.7	34.4

### Clustering of treatment-seeking behaviours

After dividing the data into three subgroups: 1-step, 2-step, and 3-step or longer (3+ steps) sequences, we conducted sequence analysis separately on each subgroup. The cluster quality measures are shown in S1 and S2 Figs. Since no strong peaks were observed in these measures, the final number of clusters was chosen as a trade-off between having a reasonably low number to maximize sample size for the subsequent analysis, with reasonably high scores on quality measures. Using this approach 10 clusters of treatment-seeking were detected: 2 clusters from the 1-step sequence subgroup, and 4 clusters each from the 2-steps and 3+ steps subgroups.

**Fig 7:**
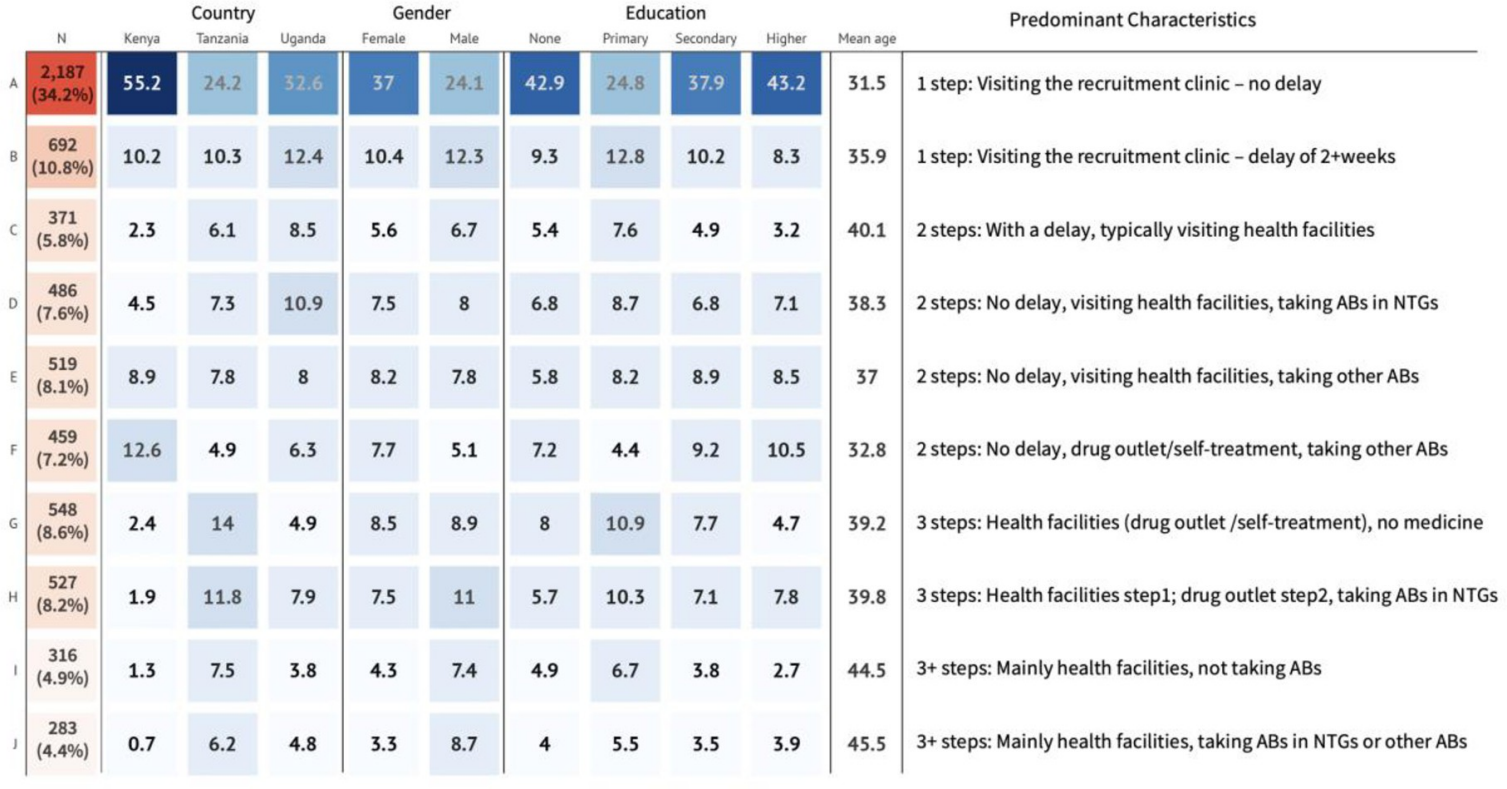
Summary of distribution, sociodemographic and the behavioral characteristics of 10 treatment-seeking clusters. Figure note: The far-left column shows the distribution of participants in each cluster (N/%). The blue cells are column percentages of cluster membership according to the country, gender and education distributions.

Fig 7 summarizes the characteristics of the 10 treatment clusters, and the distribution of behavioural characteristics of these clusters by country, gender, education, and age. Further detail is provided in S3 Fig. The most common type of cluster was to attend the recruitment clinic without delay or taking ABs (type A). After that, shorter 1-step sequences were more common, dominated by visits to health facilities. Sequences involving drug outlets (F, G,H) involved a mixture of taking ABs or other medicines. Longer sequences were more likely to involve visits to drug outlets. As might be expected from the process mining analysis, Kenyan patients were less likely to belong to more complex pathways than those from Tanzania or Uganda. They were also proportionally more likely to belong to cluster F (associated with visiting drug outlets, and taking ABs not in NTGs). Tanzanian patients were the most likely to belong to 3 or 3+ step clusters. Higher proportions of males belonged to the clusters G-J, indicating they were more likely to have complex treatment-seeking pathways. The clusters with the highest mean age were also the ones with the ongest pathways (I & J). There did not seem to be a clear relationship between education and cluster membership.

### Associations between treatment-seeking, AB use and MDR

The next stage of the analysis uses a smaller subset of patients with complete data on MDR (n = 1,946). Patients excluded at this stage were more likely to be from Tanzania or Uganda, to be older, male, and have primary education (S9 Table). Table 2 shows bivariate associations between patient characteristics and whether they had an MDR UTI or not. Patients with MDR UTI were more likely to be from Uganda and Tanzania than Kenya; to be male, older, and have none or primary school education only. Patients with MDR UTIs were also more likely to have reported at least a 2-week delay in accessing healthcare, to have had 3+ treatment-seeking steps; accessed a healthcare facility as their first step; and using ABs in their pathway. Notably, AB use in the previous 6 months was not associated with higher likelihood of MDR UTI. We repeated the bivariate analysis with MDR UTI stratifying by country (S10 Table). Some relationships were consistent across settings, but others varied. For example, in Kenya and Tanzania, males had higher levels of MDR UTI than females, but the gender relationship was reversed in Uganda. Middle-aged individuals (either 45–54 or 55–64 years) had the highest levels of MDR UTI across all 3 countries. There was no consistent educational gradient in MDR UTI. The association between delays in seeking treatment, higher treatment steps, more AB use, and MDR UTI was stronger in Tanzania and Uganda than Kenya.

**Table 2:** Sociodemographic, contextual and behavioural factors and their bivariate association with MDR.

		MDR- n(%)	MDR+ n(%)	*χ*^2^, p value
**Country**	Kenya	553 (66.2)	282 (33.8)	
	Tanzania	272 (40.5)	400 (59.5)	117.6 (p<0.001)
	Uganda	189 (43.1)	250 (56.9)	
**Gender**	Male	115 (41.5)	162 (58.5)	14.0 (p<0.001)
	Female	899 (53.9)	770 (46.1)	
**Age**	<25	298 (56.7)	228 (43.3)	27.3 (p<0.001)
	25–34	372 (55.3)	301 (44.7)	
	35–44	152 (52.6)	137 (47.4)	
	45–54	77 (44.0)	98 (56.0)	
	55–64	33 (37.1)	56 (62.9)	
	65+	82 (42.3)	112 (57.7)	
**Education**	None	131 (46.5)	151 (53.5)	56.4 (p<0.001)
	Primary	280 (42.6)	378 (57.4)	
	Secondary	383 (57.7)	281 (42.3)	
	Higher	220 (64.3)	122 (35.7)	
**Delay**	Less than 2 weeks	817 (55.1)	666 (44.9)	21.7 (p<0.001)
	More than 2 weeks	197 (42.5)	266 (57.5)	
**Treatment steps**	1(straight to clinic)	557 (56.5)	428 (43.5)	27.9 (p<0.001)
	2	292 (52.3)	266 (47.7)	
	3+	165 (40.9)	238 (59.1)	
**First care accessed**	Recruitment clinic	557 (56.5)	428 (43.5)	20.1 (p<0.001)
	Clinic	309 (45.5)	370 (54.5)	
	Pharmacy/drug shop	79 (54.5)	66 (45.5)	
	Self-treatment	69 (50.4)	68 (49.6)	
**Any AB use in pathway**	No	781 (54.1)	663 (45.9)	8.5 (p = 0.004)
	Yes	233 (46.4)	269 (53.6)	
**Any AB use in past 6 months**	No	425 (52.9)	379 (47.1)	0.3 (p = 0.608)
	Yes	589 (51.6)	553 (48.4)	

In Table 3, we go on to investigate the relationship between pathway clusters and MDR UTI using multivariable logistic regression, while adjusting for age, sex and education, and stratifying by country (Table 3). Model 1 shows the unadjusted odds ratios for MDR UTI according to pathway clusters, and model 2 shows the same odds ratios when mutually adjusted for age, sex and education. In Kenya and Tanzania there is no association between pathway clusters representing treatment seeking and AB use and risk of MDR UTI. In Uganda, clusters B, C, and I-J have significantly higher odds of MDR than those in cluster A. After controlling for age, gender and education (model 2), those in clusters I-J (representing longer, 3-step pathways) still had significantly higher odds of MDR. In models combining all three countries, there is no association between treatment pathways, AB use, individual factors and MDR UTI once country context is controlled for.

**Table 3:** Odds ratios for multi-drug resistant UTI pathogens according to treatment seeking, sociodemographic factors and country.

	Kenya (n = 835)		Tanzania (n = 672)		Uganda (n = 439)	
	Model 1 OR (95% CI)	Model 2 OR (95% CI)	Model 1 OR (95% CI)	Model 2 OR (95% CI)	Model 1 OR (95% CI)	Model 2 OR (95% CI)
Treatment cluster (ref:A)						
B	1.17(0.69–2.00)	1.09(0.63–1.87)	0.94(0.54–1.66)	0.87(0.49–1.56)	**2.06(1.01–4.19)**	1.66(0.79–3.51)
C	0.72(0.25–2.06)	0.73(0.25–2.09)	1.11(0.56–2.17)	1.07(0.54–2.14)	**2.29(1.09–4.80)**	1.72(0.78–3.8)
D	0.60(0.27–1.36)	0.50(0.21–1.16)	0.85(0.41–1.74)	0.80(0.38–1.70)	1.45(0.73–2.86)	1.27(0.63–2.57)
E	0.70(0.40–1.25)	0.64(0.36–1.15)	0.91(0.50–1.67)	0.86(0.46–1.59)	1.55(0.77–3.14)	1.31(0.62–2.76)
F	1.12(0.73–1.72)	1.12(0.72–1.74)	0.65(0.31–1.36)	0.59(0.28–1.25)	2.18(0.95–4.99)	2.06(0.88–4.79)
G	1.34(0.42–4.28)	1.23(0.37–4.04)	0.89(0.53–1.50)	0.83(0.48–1.41)	0.90(0.38–2.11)	0.64(0.25–1.60)
H	0.47(0.13–1.68)	0.45(0.12–1.66)	1.48(0.83–2.64)	1.45(0.80–2.65)	1.76(0.82–3.80)	1.30(0.56–2.99)
I + J combined	0.21(0.03–1.66)	0.16(0.02–1.32)	1.18(0.69–2.04)	1.05(0.59–1.88)	**3.94(1.76–8.86)**	**2.86(1.2–6.83)**
**Gender (ref:Female)**						
Male		0.87(0.50–1.53)		**0.53(0.33–0.85)**		1.44(0.77–2.67)
**Age (ref:<25)**						
25–34		1.29(0.91–1.83)		1.23(0.75–2.00)		1.14(0.70–1.85)
35–44		1.31(0.81–2.13)		0.73(0.43–1.26)		1.42(0.74–2.71)
45–54		**2.24(1.09–4.60)**		0.78(0.43–1.43)		1.36(0.70–2.66)
55–64		1.83(0.52–6.50)		1.20(0.62–2.29)		1.66(0.49–5.62)
65+		1.34(0.53–3.37)		0.83(0.46–1.49)		0.89(0.29–2.71)
**Education (ref:Higher)**						
None		1.09(0.28–4.23)		0.83(0.53–1.32)		**1.75(1.03–2.99)**
Primary		0.95(0.25–3.59)		0.84(0.48–1.50)		1.15(0.62–2.11)
Secondary+		0.76(0.20–2.91)		0.50(0.22–1.11)		1.63(0.75–3.56)

NB. Bold indicates estimates where 95% CIs do not cross 1.

## Discussion

This study used process mining and sequence analysis to characterise patterns of treatment-seeking behaviour among adult UTI outpatients in Kenya, Tanzania, and Uganda, and to investigate associations between those behavioural patterns and MDR UTIs. In all three countries treatment behaviour was dominated by visits to healthcare facilities. Many patients had apparent treatment failures necessitating repeat visits to clinics, which were associated with higher rates of AB use. While Kenyan patients typically had shorter pathways, they were also more likely to consult with pharmacists and drug sellers, and to take ABs not recommended for UTI in the Kenyan National Treatment Guidelines. The observed relationship between health behaviours, AB use and likelihood of MDR UTI was weak and inconsistent. In Uganda, where over half of UTI patients had MDR pathogens, patients with longer pathways, and who used more ABs recently had higher rates of MDR UTI, but this was not the case in Kenya or Tanzania. Thus, the main message is that individuals with longer treatment seeking pathways and repeat clinic visits are also more likely to have higher AB use. However, treatment pathways and past AB use are not consistently correlated with a higher risk of MDR UTI in this sample. This could be related to selection processes present in our data, or points to other contextual factors (discussed below) which contributing to the risk of developing MDR infections.

The previous, predominantly qualitative literature on the African healthcare landscape has emphasised the plurality of treatment seeking behaviours, high prevalence of self-treatment, diversity of treatment providers and parallel systems of allopathic and traditional/herbal medicine [9, 37–39]. Our study presents a somewhat different picture, where the majority behaviour in all three countries was to seek help with formal medical facilities. This is likely due to the sampling bias inherent in our study design. By recruiting clinic attendees, we selected those who are most likely to visit those facilities and missed those who sought self-treatment. On the other hand, we also may include more patients whose self-treatments had failed and who subsequently sought help from health providers. It is difficult to know how much these two selection biases offset one another.

Nevertheless, despite this acknowledged bias, we argue that the results for aim 1 highlight an often-overlooked truth about UTI care in LMICs. That is, that one of the most important complexities experienced by patients is difficulty treating UTI infections efficiently through the public healthcare system. Thus, common infections are not necessarily simple to resolve. Problems of access to care may promote medical pluralism out of necessity, resulting in more costs for patients and the healthcare system, more medicines taken unnecessarily, which further fuels the ABR crisis. Health system weaknesses are pressured further by steadily increasing levels of ABR, which make those apparently ‘simple’ infections more difficult to treat.

Among patients who took ABs to treat their UTI symptoms, the majority (two thirds) did not have a microbiologically confirmed UTI. It may be that the ‘UTI negative’ patients in our study were suffering with bacterial conditions with similar symptomatic profiles such as pelvic inflammatory disease. Regardless of this, it suggests that their AB use is not sufficiently targeted for the condition they have and this may further fuel ABR development, making it even harder to treat bacterial conditions effectively. This underlines the need for greater availability of accessible, rapid diagnostic tools [40], which could target appropriate AB use and help to slow the growth of ABR.

It is well established that excessive AB use is a driver of ABR at individual and community scales [6], and thus, we expected to find a correlation between longer pathways, higher levels of AB use, and higher rates of MDR in individual patients. Untangling temporality or cause and effect between AB use and MDR infections is complex, and where ABR is highly prevalent the two factors likely operate in a reinforcing cycle. Associations between treatment complexity and MDR could be bi-directional or explained by unmeasured factors (e.g. upstream drivers/risk factors for both complex treatment seeking and ABR). In this study, the recent treatment seeking period might be a few days, or weeks, making it less likely that that patient’s recent AB use has directly influenced the pathogen population that is responsible for their infection, or the carriage population of the bacteria in the host that may be the source of the infection (i.e. faecal carriage to urinary tract transmission). Thus, any link between recent AB use and MDR UTI is likely to be explained by primary resistance: that is, a patient acquiring an MDR UTI at the outset, and struggling to treat it, end up taking more treatment steps and more ABs.

It is also worth noting that we only observe a direct association between recent AB use and MDR in Uganda. Given that the prevalence of MDR UTI in Uganda sample was high (57%) this suggests that at least, in some settings, higher MDR might be driving longer and more complex pathways, and thus higher costs and suffering for healthcare systems and patients. We recommend further research on the longitudinal dynamic between ABR and treatment seeking, that quantify the consequences of AMR for health systems and individual’s treatment seeking behaviour.

The results highlight the importance of spatial context when considering relationships with MDR UTI. The inclusion of variables which proxy for context (e.g. country) account for more variation in MDR than individual factors or behaviours. Further, the relationships between treatment seeking and MDR UTI varied by place. There are many reasons why geographic, social and cultural context should be important [12]. For example, the more complex pathways we observe in Tanzania and Uganda may be a function of the shared structure of the healthcare system in those countries, or related to geographic features of the sites, which affect healthcare accessibility. The recruitment sites in Tanzania and Uganda were more rural, whereas the majority of the Kenyan samples are from the capital Nairobi. Likewise, spatial patterning of ABR is influenced by processes of bacterial evolution and transmission, affected by shared structural drivers such as sanitation [41–43]. It is also noteworthy that we do not observe consistent relationships between individual factors such as education and gender and MDR UTI across country settings. This raises the question of whether the AMR nexus, its social patterning, and social inter-relations is contextually specific, or whether there are generalizable relationships to identify. For example, whether there are measurable area- factors which operate as upstream drivers of both treatment seeking and ABR, such as poverty [44]. Future studies using HATUA data will explore these issues.

This study is novel in bridging several methodological and disciplinary divides. We bring together theoretically grounded understandings of patient behaviours in LMICs, generated largely from qualitative work [13,22], combine these with empiric diagnostic data, and analyse these using statistical and data-driven computer science techniques, typically used to derive patient flows from electronic health records in high-income countries. Thus, this is the first application of process mining to incorporate the breadth of formal and non-formal providers in LMICs. We also applied sequence analysis to take account of complex sequencing or different elements of pathways and cluster patients together for further analysis. We were inevitably limited by the self-reported nature of the data and the structure of the questionnaire used to collect them. One opportunity not exploited here is the inclusion of time stamps in sequences (these were unavailable in the HATUA dataset), thus we assumed all steps were equally spaced in time, which may hide important aspects. Future studies in LMICs could try to draw on existing medical records systems where available or use digital traces to construct an understanding of systems of patient flow which take account of complexity and non-formal healthcare providers.

This study has some other limitations. The linked quantitative patient sample was representative of adults attending mainly public outpatient services with UTI-like symptoms. It is likely those sampled comprised people more likely to attend clinics, thus use of other providers may be underestimated. The sample for aim 2 was restricted to those with microbiologically confirmed UTI hence is composed of people with characteristics that make UTI identification more likely (higher proportions from some country contexts, more women and those of lower education). However, patient populations are an important subgroup for possible interventions, and UTI is both prevalent and commonly treated with ABs in this region. Other data collected by the HATUA Consortium used community focus groups, and many of the same themes -–of repeat clinic visits and challenges in obtaining appropriate medications–emerged there [10]. There were also proportionally more respondents from urban higher-level referral facilities in Kenya compared to the other countries. Our decision to stratify the clustering algorithm on the number of steps would have been more robust if we had preliminary data on which to test the analysis strategy. Finally, we analysed a simple set of individual level predictors, with a focus on behavioural antecedents of clinic attendance, but our own qualitative work [10,11] suggests that a richer set of covariates at individual, household and community are necessary to understand the burden of ABR, as well as longitudinal follow-up. This will be addressed in future HATUA studies which take account of the inter-related and multi-scalar nature of ABR drivers.

## Conclusion

This novel interdisciplinary mixed-methods study investigates treatment seeking, AB use and risk of MDR UTI in East Africa. The results highlight the complexities of treatment-seeking for UTI care in East Africa, and the potential for higher rates of ABR to complicate patient treatment seeking. In Tanzania and Uganda, where more than half of UTI patients surveyed have MDR UTIs and overall prevalence of ABR is high, repeated treatment failures are an inevitable consequence of patients seeking to alleviate their symptoms. Such frustrating and expensive cycles of treatment place a heavy burden on already struggling individuals and healthcare structures, and feed into a vicious cycle of ineffective AB use and further selection pressure for ABR in pathogen populations. Solutions for arresting this cycle are likely multifaceted, including improving access to rapid diagnostic and susceptibility testing for UTIs, appropriate AB treatment, effective AB stewardship, and addressing the upstream drivers of infection, through behavioural factors like hygiene and environmental determinants such as sanitation.

## Supporting information

S1 Checklist Questionnaire inclusivity in global research. (DOCX)

S2 Checklist STROBE checklist. (DOCX)

S1 Table Patient recruitment sites in Kenya, Tanzania, and Uganda. (DOCX)

S2 Table Antibiotics considered for MDR calculations. (DOCX)

S3 Table List of antibiotics mentioned by patients and whether they are recommended for use for treating UTI according to country National Treatment Guidelines (NTG). (DOCX)

S4 Table Fig 3 image attributions. (DOCX)

S5 Table Characteristics of the patient sample used for the two stages of the analysis. (DOCX)

S6 Table Kenya: Characteristics of the patient sample used for the two stages of the analysis. (DOCX)

S7 Table Tanzania: Characteristics of the patient sample used for the two stages of the analysis. (DOCX)

S8 Table Uganda: Characteristics of the patient sample used for the two stages of the analysis. (DOCX)

S9 Table Characteristics of patients included and excluded from the analysis samples. (DOCX)

S10 Table Bivariate associations between patient characteristics and MDR, stratified by country. (DOCX)

S1 Fig Clustering quality for subgroup of sequences including 2 steps. (DOCX)

S2 Fig Clustering quality for subgroup of sequences including 3 or more steps. (DOCX)

S3 Fig Characteristics of the 10 clusters detected by sequence analysis. (DOCX)

S1 Data Data file. (XLSX)
