# Supplementary material for: Treatment seeking behaviours, antibiotic use and relationships to multi-drug resistance: A study of urinary tract infection patients in Kenya, Tanzania and Uganda
Source: PLOS Glob Public Health. 2024 Feb 16;4(2):e0002709. doi: 10.1371/journal.pgph.0002709 (PMC10871516; doi:10.1371/journal.pgph.0002709)
Supplement: S1 Table — (DOCX) [file pgph.0002709.s003.docx]

| **Country/site** | **Number of facilities** | **Source of funding** | **Levels recruited from^1^** |
| --- | --- | --- | --- |
| **Kenya** |  |  |  |
| Makueni | 1 | Public | 5 |
| Nairobi | 4 | Public and private | 3-5, National |
| Nanyuki | 1 | Public | 4 |
| **Tanzania** |  |  |  |
| Kilimanjaro/Moshi | 3 | Public and private | 2,3, and 5 |
| Mbeya | 2 | Public and private | 3 and 4 |
| Mwanza | 5 | Public and private | 2,3, and 5 |
| **Uganda** |  |  |  |
| Mbarara | 3 | Public | 3 and 5 |
| Nakapiripirit | 3 | Public | 2 and 3 |
| Nakasongola | 3 | Public and private | 3 and 4 |

^1^ Levels of facilities are identified in each country following the Kenya Health Policy 2014-2013 (<http://publications.universalhealth2030.org/uploads/kenya_health_policy_2014_to_2030.pdf>); Tanzanian Health Sector Strategic Plan, <https://p4h.world/en/node/11813#:~:text=2026%20%7C%20P4H%20Network-,Tanzania%20fifth%20health%20sector%20strategic%20plan%20(HSSPV)%2D%202021%2D2026,coverage%20(UHC)%20by%202030> and the Ugandan Hospital and Health Centre IV census survey (<http://library.health.go.ug/publications/health-infrastructure/uganda-hospital-and-health-centre-iv-census-survey>)
