# Supplementary material for: Treatment seeking behaviours, antibiotic use and relationships to multi-drug resistance: A study of urinary tract infection patients in Kenya, Tanzania and Uganda
Source: PLOS Glob Public Health. 2024 Feb 16;4(2):e0002709. doi: 10.1371/journal.pgph.0002709 (PMC10871516; doi:10.1371/journal.pgph.0002709)
Supplement: S2 Table — (DOCX) [file pgph.0002709.s004.docx]

**Table S2.** Antibiotics considered for MDR calculations.

| **Gram negative** | **Amoxicillin/**  **clavulanate**  **(AMC)** | **Ampicillin**  **(AMP)** | **Ceftazidime/**  **Ceftriaxone**  **(CAZ/CRO)** | **Ciprofloxacin**  **(CIP)** | **Gentamicin**  **(GEN)** | **Nitrofurantoin**  **(NIT)** | **Trimethoprim**  **(TMP)** |  | |
| --- | --- | --- | --- | --- | --- | --- | --- | --- | --- |
| ***E. coli*** | AMC | AMP | CAZ/CRO | CIP | GEN | NIT | TMP |  | |
| ***Shigella* spp.** | AMC | AMP | CAZ/CRO | CIP | GEN | NIT | TMP |  | |
| ***Proteus* spp.** | AMC | AMP | CAZ/CRO | CIP | GEN | NIT | TMP |  | |
| ***Salmonella* spp.** | AMC | AMP | CAZ/CRO | CIP | GEN | NIT | TMP |  | |
| ***Serratia* spp.** | - | - | CAZ/CRO | CIP | GEN | NIT | TMP |  | |
| ***Klebsiella* spp.** | AMC | - | CAZ/CRO | CIP | GEN | NIT | TMP |  | |
| ***Citrobacter* spp.** | **-** | - | CAZ/CRO | CIP | GEN | NIT | TMP |  | |
| ***Enterobacter* spp.** | - | - | CAZ/CRO | CIP | GEN | NIT | TMP |  | |
| ***Morganella* spp.** | **-** | **-** | CAZ/CRO | CIP | GEN | NIT | TMP |  | |
| ***Pantoea* spp.** | - | - | CAZ/CRO | CIP | GEN | NIT | TMP |  | |
| ***Providencia* spp.** | - | - | CAZ/CRO | CIP | GEN | NIT | TMP |  | |
| ***Acinetobacter* spp.** | - | - | CAZ/CRO | CIP | GEN | - | TMP |  | |
| ***Pseudomonas* spp.** | - | - | CAZ | CIP | GEN | - | - |  | |
| **Gram positive** | **Cefoxitin**  **(FOX)** | **Erythromycin**  **(ERY)** | **Linezolid**  **(LNZ)** | **Ciprofloxacin**  **(CIP)** | **Gentamicin**  **(GEN)** | **Nitrofurantoin**  **(NIT)** | **Trimethoprim**  **(TMP)** | **Tetracycline**  **(TCY)** | **Vancomycin**  **(VAN)** |
| ***Staphylococcus* spp.** | FOX | ERY | - | CIP | GEN | NIT | TMP | TCY | - |
| ***Enterococcus* spp.** | - | ERY | LNZ | CIP | - | NIT | - | TCY | VAN |
| ***Streptococcus* spp.** | **-** | ERY | LNZ | - | - | NIT | - | TCY | VAN |
