# Supplementary material for: Treatment seeking behaviours, antibiotic use and relationships to multi-drug resistance: A study of urinary tract infection patients in Kenya, Tanzania and Uganda
Source: PLOS Glob Public Health. 2024 Feb 16;4(2):e0002709. doi: 10.1371/journal.pgph.0002709 (PMC10871516; doi:10.1371/journal.pgph.0002709)
Supplement: S3 Table — (DOCX) [file pgph.0002709.s005.docx]

**Table S3.** List of antibiotics mentioned by patients and whether they are recommended for use for treating UTI according to country National Treatment Guidelines (NTG)

|  | **Kenya NTG^1^**  (adult outpatients- not admitted to hospital) | **Tanzania NTG^2^**  (adult outpatients- not admitted to hospital) | **Uganda NTG^3^**  (adult outpatients- not admitted to hospital) |
| --- | --- | --- | --- |
| Amoxicillin | a, b |  | c |
| Amoxicillin/ Clavulanic Acid (Amoxiclav) |  | b, c |  |
| Ampicillin | a |  | b, c |
| Ampicillin-Cloxacillin (Ampiclox) |  |  |  |
| Azithromycin (Azuma) |  |  |  |
| Ceftriaxone |  |  | b, c |
| Cefuroxime |  |  |  |
| Cephalexine |  |  |  |
| Ciprofloxacin |  | a, b | a, b |
| Cotrimoxozole (Septrine) | a, b |  |  |
| Doxycycline |  |  |  |
| Erythromycin |  |  |  |
| Gentamicin |  |  | b, c |
| Levofloxacin |  |  |  |
| Metronidazole (Flagyl) |  |  |  |
| Nitrofurantoin | a |  | a, c |
| Tetracycline |  |  |  |
| Tinidazole |  |  |  |

a= uncomplicated lower UTI

b= uncomplicated upper UTI (includes for Uganda, first and second line options)

c= treatments recommended for pregnant women only

**^1^** [**^http://guidelines.health.go.ke:8000/media/Clinical_Guidelines_Vol_II_Final.pdf^**](http://guidelines.health.go.ke:8000/media/Clinical_Guidelines_Vol_II_Final.pdf)

**^2^** [**^https://hssrc.tamisemi.go.tz/storage/app/uploads/public/5ab/e9b/b21/5abe9bb216267130384889.pdf^**](https://hssrc.tamisemi.go.tz/storage/app/uploads/public/5ab/e9b/b21/5abe9bb216267130384889.pdf)

**^3^** [**^https://www.prb.org/wp-content/uploads/2018/05/Uganda-Clinical-Guidelines-2016-National-Guidelines-for-Management-of-Common-Conditions.pdf^**](https://www.prb.org/wp-content/uploads/2018/05/Uganda-Clinical-Guidelines-2016-National-Guidelines-for-Management-of-Common-Conditions.pdf)
