# Supplementary material for: Treatment seeking behaviours, antibiotic use and relationships to multi-drug resistance: A study of urinary tract infection patients in Kenya, Tanzania and Uganda
Source: PLOS Glob Public Health. 2024 Feb 16;4(2):e0002709. doi: 10.1371/journal.pgph.0002709 (PMC10871516; doi:10.1371/journal.pgph.0002709)
Supplement: S4 Table — (DOCX) [file pgph.0002709.s006.docx]

**Table S4:** Figure 3 Image attributions

|  | Page URL | Attribution |
| --- | --- | --- |
| Human icon | https://commons.wikimedia.org/wiki/File:Person_icon_BLACK-01.svg | MCruz (WMF), CC BY-SA 4.0 <https://creativecommons.org/licenses/by-sa/4.0>, via Wikimedia Commons |
| Black Clock | https://commons.wikimedia.org/wiki/File:OOjs_UI_icon_clock.svg | OOjs UI Team and other contributors, MIT <http://opensource.org/licenses/mit-license.php>, via Wikimedia Commons |
| White Clock | https://commons.wikimedia.org/wiki/File:Clock_(CoreUI_Icons_v1.0.0).svg | CoreUI, CC BY 4.0 <https://creativecommons.org/licenses/by/4.0>, via Wikimedia Commons |
| Hospital | https://commons.wikimedia.org/wiki/File:Hospital_Icon.png | Upload12345, CC BY-SA 4.0 <https://creativecommons.org/licenses/by-sa/4.0>, via Wikimedia Commons |
| House | https://commons.wikimedia.org/wiki/File:BSicon_HOUSE.svg | P999, Public domain, via Wikimedia Commons |
| pill | https://commons.wikimedia.org/wiki/File:Tabler-icons_pill.svg | Tabler, MIT <http://opensource.org/licenses/mit-license.php>, via Wikimedia Commons |
