# Supplementary material for: Treatment seeking behaviours, antibiotic use and relationships to multi-drug resistance: A study of urinary tract infection patients in Kenya, Tanzania and Uganda
Source: PLOS Glob Public Health. 2024 Feb 16;4(2):e0002709. doi: 10.1371/journal.pgph.0002709 (PMC10871516; doi:10.1371/journal.pgph.0002709)
Supplement: S5 Table — (DOCX) [file pgph.0002709.s007.docx]

**Table S5:** Characteristics of the patient sample used for the two stages of the analysis

|  |  | **Analysis sample: Pathway characteristics** | | **Analysis sample: Associations with MDR** | |
| --- | --- | --- | --- | --- | --- |
|  |  | **N** | **%** | **N** | **%** |
| **Country** | Kenya | 1,591 | 24.9 | 835 | 42.9 |
|  | Tanzania | 3,046 | 47.7 | 672 | 34.5 |
|  | Uganda | 1,751 | 27.4 | 439 | 22.6 |
| **Age** | <25 | 1,695 | 26.5 | 526 | 27.0 |
|  | 25-34 | 2,114 | 33.1 | 673 | 34.6 |
|  | 35-44 | 1,021 | 16.0 | 175 | 9.0 |
|  | 45-54 | 632 | 9.9 | 89 | 4.6 |
|  | 55-64 | 373 | 5.8 | 289 | 14.9 |
|  | 65+ | 553 | 8.7 | 194 | 10.0 |
| **Gender** | Male | 1,367 | 21.4 | 277 | 14.2 |
|  | Female | 5,021 | 78.6 | 1,669 | 85.8 |
| **Education** | None | 965 | 15.1 | 282 | 14.5 |
|  | Primary | 2,525 | 39.5 | 658 | 33.8 |
|  | Secondary | 1,984 | 31.1 | 664 | 34.1 |
|  | Higher | 914 | 14.3 | 342 | 17.6 |
| **Treatment steps** | 1(straight to clinic) | 2,879 | 45.1 | 985 | 50.6 |
|  | 2 | 1,835 | 28.7 | 558 | 28.7 |
|  | 3+ | 1,674 | 26.2 | 403 | 20.7 |
| **AB use in pathway** | No | 4,505 | 70.5 | 1,444 | 74.2 |
|  | Yes | 1,883 | 29.5 | 502 | 25.8 |
| **AB use past 6m** | No | 2,482 | 38.9 | 804 | 41.3 |
|  | Yes | 3,906 | 61.1 | 1,142 | 58.7 |
| **UTI status** | Negative | 4,194 | 65.7 | 0 | 0 |
|  | Positive | 2,194 | 34.3 | 1,946 | 100 |
| **MDR status** | Negative |  |  | 1,014 | 52.1 |
|  | Positive |  |  | 932 | 47.9 |
| **TOTAL** |  | 6,388 | 100.0 | 1,946 | 100.0 |
