# Supplementary material for: Treatment seeking behaviours, antibiotic use and relationships to multi-drug resistance: A study of urinary tract infection patients in Kenya, Tanzania and Uganda
Source: PLOS Glob Public Health. 2024 Feb 16;4(2):e0002709. doi: 10.1371/journal.pgph.0002709 (PMC10871516; doi:10.1371/journal.pgph.0002709)
Supplement: S6 Table — (DOCX) [file pgph.0002709.s008.docx]

**Table S6:** Kenya: characteristics of the patient sample used for the two stages of the analysis

|  |  | **Analysis sample: Pathway characteristics** | | **Analysis sample: Associations with MDR** | |
| --- | --- | --- | --- | --- | --- |
|  |  | **N** | **%** | **N** | **%** |
| **Age** | <25 | 433 | 27.2 | 245 | 29.3 |
|  | 25-34 | 753 | 47.3 | 389 | 46.6 |
|  | 35-44 | 254 | 16.0 | 39 | 4.7 |
|  | 45-54 | 85 | 5.3 | 11 | 1.3 |
|  | 55-64 | 25 | 1.6 | 123 | 14.7 |
|  | 65+ | 41 | 2.6 | 28 | 3.4 |
| **Gender** | Male | 258 | 16.2 | 66 | 7.9 |
|  | Female | 1,333 | 83.8 | 769 | 92.1 |
| **Education** | None | 19 | 1.2 | 11 | 1.3 |
|  | Primary | 195 | 12.3 | 99 | 11.9 |
|  | Secondary | 889 | 55.9 | 460 | 55.1 |
|  | Higher | 488 | 30.7 | 265 | 31.7 |
| **Treatment steps** | 1(straight to clinic) | 1,041 | 65.4 | 574 | 68.7 |
|  | 2 | 450 | 28.3 | 224 | 26.8 |
|  | 3+ | 100 | 6.3 | 37 | 4.4 |
| **AB use in pathway** | No | 1,303 | 81.9 | 690 | 82.6 |
|  | Yes | 288 | 18.1 | 145 | 17.4 |
| **AB use past 6m** | No | 758 | 47.6 | 370 | 44.3 |
|  | Yes | 833 | 52.4 | 465 | 55.7 |
| **UTI status** | Negative | 716 | 45.0 | 0 | 0 |
|  | Positive | 875 | 55.0 | 835 | 100 |
| **MDR status** | Negative |  |  | 553 | 66.2 |
|  | Positive |  |  | 282 | 33.8 |
| **TOTAL** |  | 1,591 | 100.0 | 835 | 100.0 |
