# Supplementary material for: Treatment seeking behaviours, antibiotic use and relationships to multi-drug resistance: A study of urinary tract infection patients in Kenya, Tanzania and Uganda
Source: PLOS Glob Public Health. 2024 Feb 16;4(2):e0002709. doi: 10.1371/journal.pgph.0002709 (PMC10871516; doi:10.1371/journal.pgph.0002709)
Supplement: S7 Table — (DOCX) [file pgph.0002709.s009.docx]

**Table S7:** Tanzania: characteristics of the patient sample used for the two stages of the analysis

|  |  | **Analysis sample: Pathway characteristics** | | **Analysis sample: Associations with MDR** | |
| --- | --- | --- | --- | --- | --- |
|  |  | **N** | **%** | **N** | **%** |
| **Age** | <25 | 675 | 22.2 | 139 | 20.7 |
|  | 25-34 | 799 | 26.2 | 140 | 20.8 |
|  | 35-44 | 469 | 15.4 | 101 | 15.0 |
|  | 45-54 | 375 | 12.3 | 78 | 11.6 |
|  | 55-64 | 278 | 9.1 | 65 | 9.7 |
|  | 65+ | 450 | 14.8 | 149 | 22.2 |
| **Gender** | Male | 832 | 27.3 | 158 | 23.5 |
|  | Female | 2,214 | 72.7 | 514 | 76.5 |
| **Education** | None | 349 | 11.5 | 113 | 16.8 |
|  | Primary | 1,678 | 55.1 | 400 | 59.5 |
|  | Secondary | 758 | 24.9 | 122 | 18.2 |
|  | Higher | 261 | 8.6 | 37 | 5.5 |
| **Treatment steps** | 1(straight to clinic) | 1,051 | 34.5 | 221 | 32.9 |
|  | 2 | 795 | 26.1 | 183 | 27.2 |
|  | 3+ | 1,200 | 39.4 | 268 | 39.9 |
| **AB use in pathway** | No | 2,056 | 67.5 | 467 | 69.5 |
|  | Yes | 990 | 32.5 | 205 | 30.5 |
| **AB use past 6m** | No | 510 | 16.7 | 139 | 20.7 |
|  | Yes | 2,536 | 83.3 | 533 | 79.3 |
| **UTI status** | Negative | 2,214 | 72.7 | 0 | 0 |
|  | Positive | 832 | 27.3 | 672 | 100 |
| **MDR status** | Negative |  |  | 272 | 40.5 |
|  | Positive |  |  | 400 | 59.5 |
| **TOTAL** |  | 3,046 | 100.0 | 672 | 100.0 |
