# Supplementary material for: Treatment seeking behaviours, antibiotic use and relationships to multi-drug resistance: A study of urinary tract infection patients in Kenya, Tanzania and Uganda
Source: PLOS Glob Public Health. 2024 Feb 16;4(2):e0002709. doi: 10.1371/journal.pgph.0002709 (PMC10871516; doi:10.1371/journal.pgph.0002709)
Supplement: S8 Table — (DOCX) [file pgph.0002709.s010.docx]

**Table S8:** Uganda: characteristics of the patient sample used for the two stages of the analysis

|  |  | **Analysis sample: Pathway characteristics** | | **Analysis sample: Associations with MDR** | |
| --- | --- | --- | --- | --- | --- |
|  |  | **N** | **%** | **N** | **%** |
| **Age** | <25 | 587 | 33.5 | 142 | 32.3 |
|  | 25-34 | 562 | 32.1 | 144 | 32.8 |
|  | 35-44 | 298 | 17.0 | 65 | 14.8 |
|  | 45-54 | 172 | 9.8 | 58 | 13.2 |
|  | 55-64 | 70 | 4.0 | 13 | 3.0 |
|  | 65+ | 62 | 3.5 | 17 | 3.9 |
| **Gender** | Male | 277 | 15.8 | 53 | 12.1 |
|  | Female | 1,474 | 84.2 | 386 | 87.9 |
| **Education** | None | 597 | 34.1 | 158 | 36.0 |
|  | Primary | 652 | 37.2 | 159 | 36.2 |
|  | Secondary | 337 | 19.2 | 82 | 18.7 |
|  | Higher | 165 | 9.4 | 40 | 9.1 |
| **Treatment steps** | 1(straight to clinic) | 787 | 44.9 | 190 | 43.3 |
|  | 2 | 590 | 33.7 | 151 | 34.4 |
|  | 3+ | 374 | 21.4 | 98 | 22.3 |
| **AB use in pathway** | No | 1,146 | 65.4 | 287 | 65.4 |
|  | Yes | 605 | 34.6 | 152 | 34.6 |
| **AB use past 6m** | No | 1,214 | 69.3 | 295 | 67.2 |
|  | Yes | 537 | 30.7 | 144 | 32.8 |
| **UTI status** | Negative | 1,264 | 72.2 | 0 | 0 |
|  | Positive | 487 | 27.8 | 439 | 100 |
| **MDR status** | Negative |  |  | 189 | 43.1 |
|  | Positive |  |  | 250 | 56.9 |
| **TOTAL** |  | 1,751 | 100.0 | 439 | 100.0 |
