# Supplementary material for: Treatment seeking behaviours, antibiotic use and relationships to multi-drug resistance: A study of urinary tract infection patients in Kenya, Tanzania and Uganda
Source: PLOS Glob Public Health. 2024 Feb 16;4(2):e0002709. doi: 10.1371/journal.pgph.0002709 (PMC10871516; doi:10.1371/journal.pgph.0002709)
Supplement: S9 Table — (DOCX) [file pgph.0002709.s011.docx]

**Table S9.** Characteristics of patients included and excluded from the analysis samples

|  |  | **Analysis sample 1** | | **Analysis sample 2 – UTI + and MDR data** | |
| --- | --- | --- | --- | --- | --- |
|  |  | **Included** | **Excluded (N%)** | **Included** | **Excluded N(%)** |
| **Country** | **Kenya** | 1,591 | 70(4.2) | 835 | 826(49.7) |
|  | **Tanzania** | 3,046 | 144(4.5) | 672 | 2518(78.9) |
|  | **Uganda** | 1,751 | 6(0.3) | 439 | 1318(75) |
| **Age** | **<25** | 1,695 | 47(2.7) | 526 | 1216(69.8) |
|  | **25-34** | 2,114 | 72(3.3) | 673 | 1513(69.2) |
|  | **35-44** | 1,021 | 27(2.6) | 289 | 759(72.4) |
|  | **45-54** | 632 | 9(1.4) | 175 | 466(72.7) |
|  | **55-64** | 373 | 5(1.3) | 89 | 289(76.5) |
|  | **65+** | 553 | 15(2.6) | 194 | 374(65.8) |
| **Gender** | **Male** | 1,367 | 42(3) | 277 | 1132(80.3) |
|  | **Female** | 5,021 | 178(3.4) | 1669 | 3530(67.9) |
| **Education** | **None** | 965 | 33(3.3) | 282 | 716(71.7) |
|  | **Primary** | 2,525 | 103(3.9) | 658 | 1970(75) |
|  | **Secondary** | 1,984 | 50(2.5) | 664 | 1370(67.4) |
|  | **Higher** | 914 | 29(3.1) | 342 | 601(63.7) |
| **TOTAL** |  | 6,388 | 220 (3.3) | 1,946 | 4,662 (70.1) |
