# Supplementary material for: Treatment seeking behaviours, antibiotic use and relationships to multi-drug resistance: A study of urinary tract infection patients in Kenya, Tanzania and Uganda
Source: PLOS Glob Public Health. 2024 Feb 16;4(2):e0002709. doi: 10.1371/journal.pgph.0002709 (PMC10871516; doi:10.1371/journal.pgph.0002709)
Supplement: S10 Table — (DOCX) [file pgph.0002709.s012.docx]

**Table S10:** Bivariate associations between patient characteristics and MDR, stratified by country

|  |  |  | MDR-  n(%) | MDR+  n(%) | $\chi^{2}$, p value |
| --- | --- | --- | --- | --- | --- |
| **Gender** | Kenya | Male | 40 (60.6) | 26 (39.4) | 0.8 (0.384) |
|  |  | Female | 513 (66.7) | 256 (33.3) |  |
|  | Tanzania | Male | 49 (31.0) | 109 (69.0) | 7.2 (0.007) |
|  |  | Female | 223 (43.4) | 291 (56.6) |  |
|  | Uganda | Male | 26 (49.1) | 27 (50.9) | 0.6 (0.427) |
|  |  | Female | 163 (42.2) | 223 (57.8) |  |
| **Age** | Kenya | <25 | 172 (70.2) | 73 (29.8) | 6.4 (0.271) |
|  |  | 25-34 | 256 (65.8) | 133 (34.2) |  |
|  |  | 35-44 | 81 (65.9) | 42 (34.1) |  |
|  |  | 45-54 | 20 (51.3) | 19 (48.7) |  |
|  |  | 55-64 | 6 (54.5) | 5 (45.5) |  |
|  |  | 65+ | 18 (64.3) | 10 (35.7) |  |
|  | Tanzania | <25 | 59 (42.4) | 80 (57.6) | 5.8 (0.331) |
|  |  | 25-34 | 54 (38.6) | 86 (61.4) |  |
|  |  | 35-44 | 49 (48.5) | 52 (51.5) |  |
|  |  | 45-54 | 34 (43.6) | 44 (56.4) |  |
|  |  | 55-64 | 22 (33.8) | 43 (66.2) |  |
|  |  | 65+ | 54 (36.2) | 95 (63.8) |  |
|  | Uganda | <25 | 67 (47.2) | 75 (52.8) | 5.3 (0.375) |
|  |  | 25-34 | 62 (43.1) | 82 (56.9) |  |
|  |  | 35-44 | 22 (33.8) | 43 (66.2) |  |
|  |  | 45-54 | 23 (39.7) | 35 (60.3) |  |
|  |  | 55-64 | 5 (38.5) | 8 (61.5) |  |
|  |  | 65+ | 10 (58.8) | 7 (41.2) |  |
| **Education** | Kenya | None | 7 (63.6) | 4 (36.4) | 2.9 (0.408) |
|  |  | Primary | 62 (62.6) | 37 (37.4) |  |
|  |  | Secondary | 298 (64.8) | 162 (35.2) |  |
|  |  | Higher | 186 (70.2) | 79 (29.8) |  |
|  | Tanzania | None | 42 (37.2) | 71 (62.8) | 2.4 (0.492) |
|  |  | Primary | 163 (40.8) | 237 (59.2) |  |
|  |  | Secondary | 48 (39.3) | 74 (60.7) |  |
|  |  | Higher | 19 (51.4) | 18 (48.6) |  |
|  | Uganda | None | 82 (51.9) | 76 (48.1) | 10.3 (0.016) |
|  |  | Primary | 55 (34.6) | 104 (65.4) |  |
|  |  | Secondary | 37 (45.1) | 45 (54.9) |  |
|  |  | Higher | 15 (37.5) | 25 (62.5) |  |
| **Delay in accessing care** | Kenya | Less than 2 weeks | 490 (66.3) | 249 (33.7) | 0.0 (0.986) |
|  |  | More than 2 weeks | 63 (65.6) | 33 (34.4) |  |
|  | Tanzania | Less than 2 weeks | 176 (41.1) | 252 (58.9) | 0.1 (0.712) |
|  |  | More than 2 weeks | 96 (39.3) | 148 (60.7) |  |
|  | Uganda | Less than 2 weeks | 151 (47.8) | 165 (52.2) | 9.6 (0.002) |
|  |  | More than 2 weeks | 38 (30.9) | 85 (69.1) |  |
| **Treatment-seeking steps** | Kenya | 1(straight to clinic) | 372 (64.8) | 202 (35.2) | 2.4 (0.297) |
|  |  | 2 | 153 (68.3) | 71 (31.7) |  |
|  |  | 3+ | 28 (75.7) | 9 (24.3) |  |
|  | Tanzania | 1(straight to clinic) | 91 (41.2) | 130 (58.8) | 1.7 (0.426) |
|  |  | 2 | 80 (43.7) | 103 (56.3) |  |
|  |  | 3+ | 101 (37.7) | 167 (62.3) |  |
|  | Uganda | 1(straight to clinic) | 94 (49.5) | 96 (50.5) | 5.8 (0.056) |
|  |  | 2 | 59 (39.1) | 92 (60.9) |  |
|  |  | 3+ | 36 (36.7) | 62 (63.3) |  |
| **First care accessed** | Kenya | Recruitment clinic | 372 (64.8) | 202 (35.2) | 5.2 (0.156) |
|  |  | Clinic | 98 (74.8) | 33 (25.2) |  |
|  |  | Pharmacy/drug shop | 44 (62.9) | 26 (37.1) |  |
|  |  | Self-treatment | 39 (65.0) | 21 (35.0) |  |
|  | Tanzania | Recruitment clinic | 91 (41.2) | 130 (58.8) | 4.0 (0.265) |
|  |  | Clinic | 135 (38.0) | 220 (62.0) |  |
|  |  | Pharmacy/drug shop | 29 (51.8) | 27 (48.2) |  |
|  |  | Self-treatment | 17 (42.5) | 23 (57.5) |  |
|  | Uganda | Recruitment clinic | 94 (49.5) | 96 (50.5) | 6.2 (0.101) |
|  |  | Clinic | 76 (39.4) | 117 (60.6) |  |
|  |  | Pharmacy/drug shop | 6 (31.6) | 13 (68.4) |  |
|  |  | Self-treatment | 13 (35.1) | 24 (64.9) |  |
| **Any AB use in pathway** | Kenya | No | 452 (65.5) | 238 (34.5) | 0.7 (0.388) |
|  |  | Yes | 101 (69.7) | 44 (30.3) |  |
|  | Tanzania | No | 197 (42.2) | 270 (57.8) | 1.6 (0.202) |
|  |  | Yes | 75 (36.6) | 130 (63.4) |  |
|  | Uganda | No | 132 (46.0) | 155 (54.0) | 2.6 (0.108) |
|  |  | Yes | 57 (37.5) | 95 (62.5) |  |
| TOTAL |  |  |  |  |  |
