# Supplementary figures and images for: Treatment seeking behaviours, antibiotic use and relationships to multi-drug resistance: A study of urinary tract infection patients in Kenya, Tanzania and Uganda
Source: PLOS Glob Public Health. 2024 Feb 16;4(2):e0002709. doi: 10.1371/journal.pgph.0002709 (PMC10871516; doi:10.1371/journal.pgph.0002709)

**S1 Fig. Clustering quality for subgroup of sequences including 2 steps**


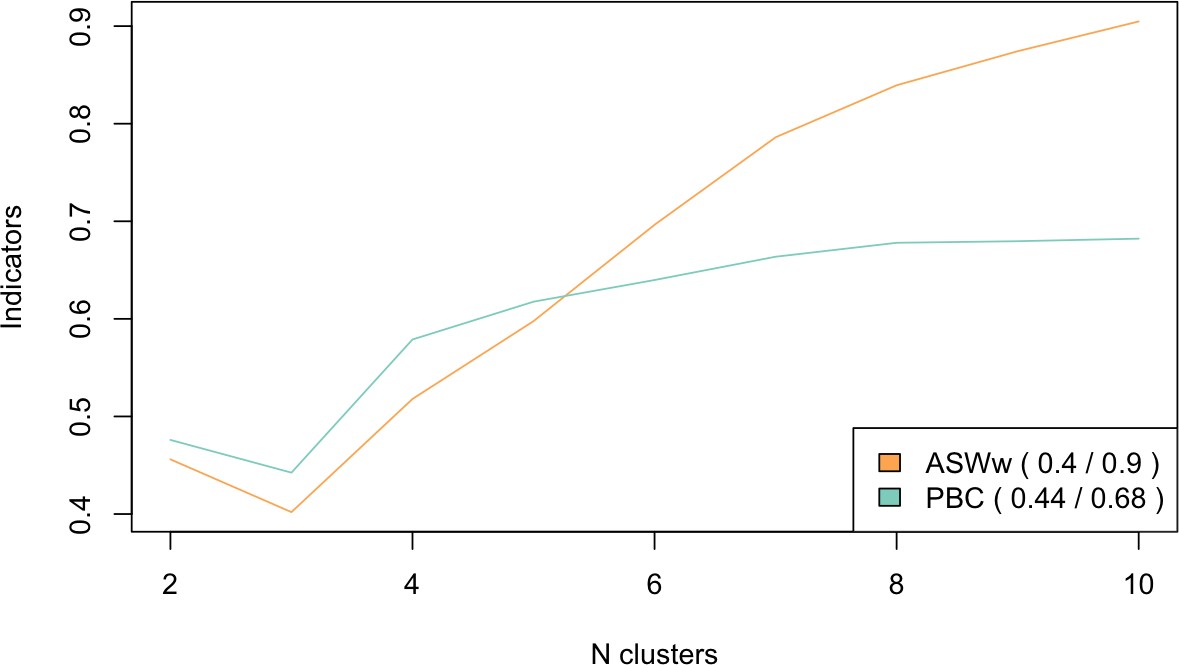

Supplement: S1 Fig — (DOCX) [file pgph.0002709.s013.docx]

**
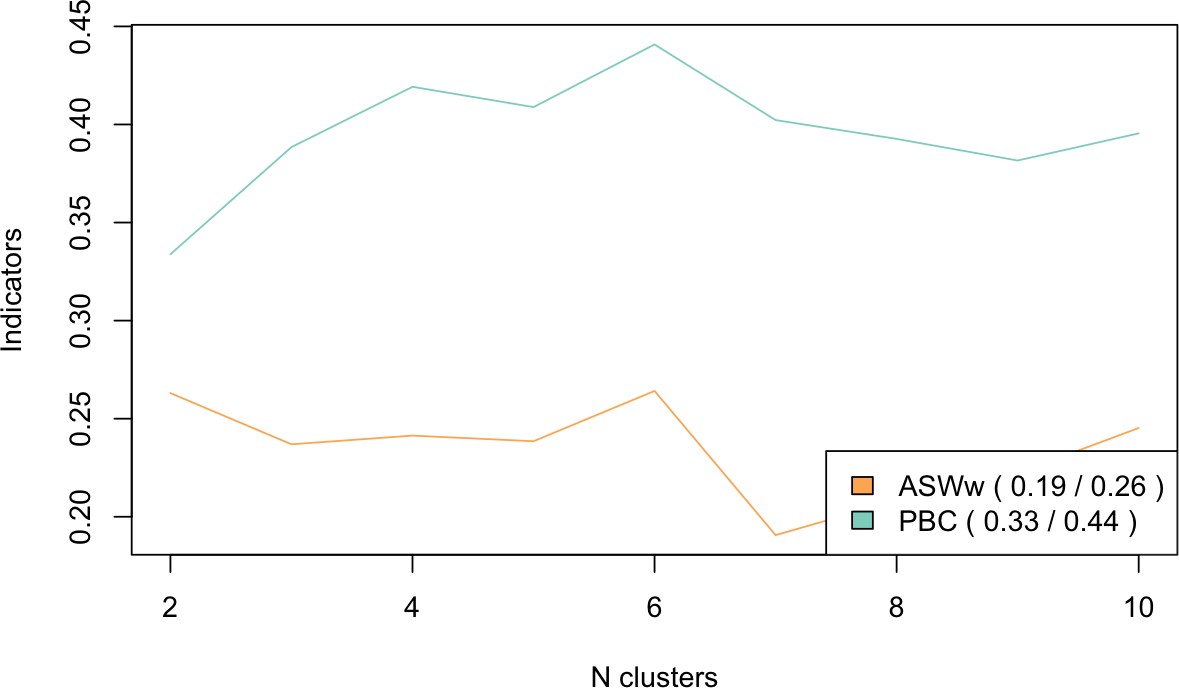
S2 Fig. Clustering quality for subgroup of sequences including 3 or more steps**

Supplement: S2 Fig — (DOCX) [file pgph.0002709.s014.docx]
