# Supplementary material for: Treatment seeking behaviours, antibiotic use and relationships to multi-drug resistance: A study of urinary tract infection patients in Kenya, Tanzania and Uganda
Source: PLOS Glob Public Health. 2024 Feb 16;4(2):e0002709. doi: 10.1371/journal.pgph.0002709 (PMC10871516; doi:10.1371/journal.pgph.0002709)
Supplement: S3 Fig — (DOCX) [file pgph.0002709.s015.docx]

**S3 Fig. Characteristics of the 10 clusters detected by sequence analysis.**


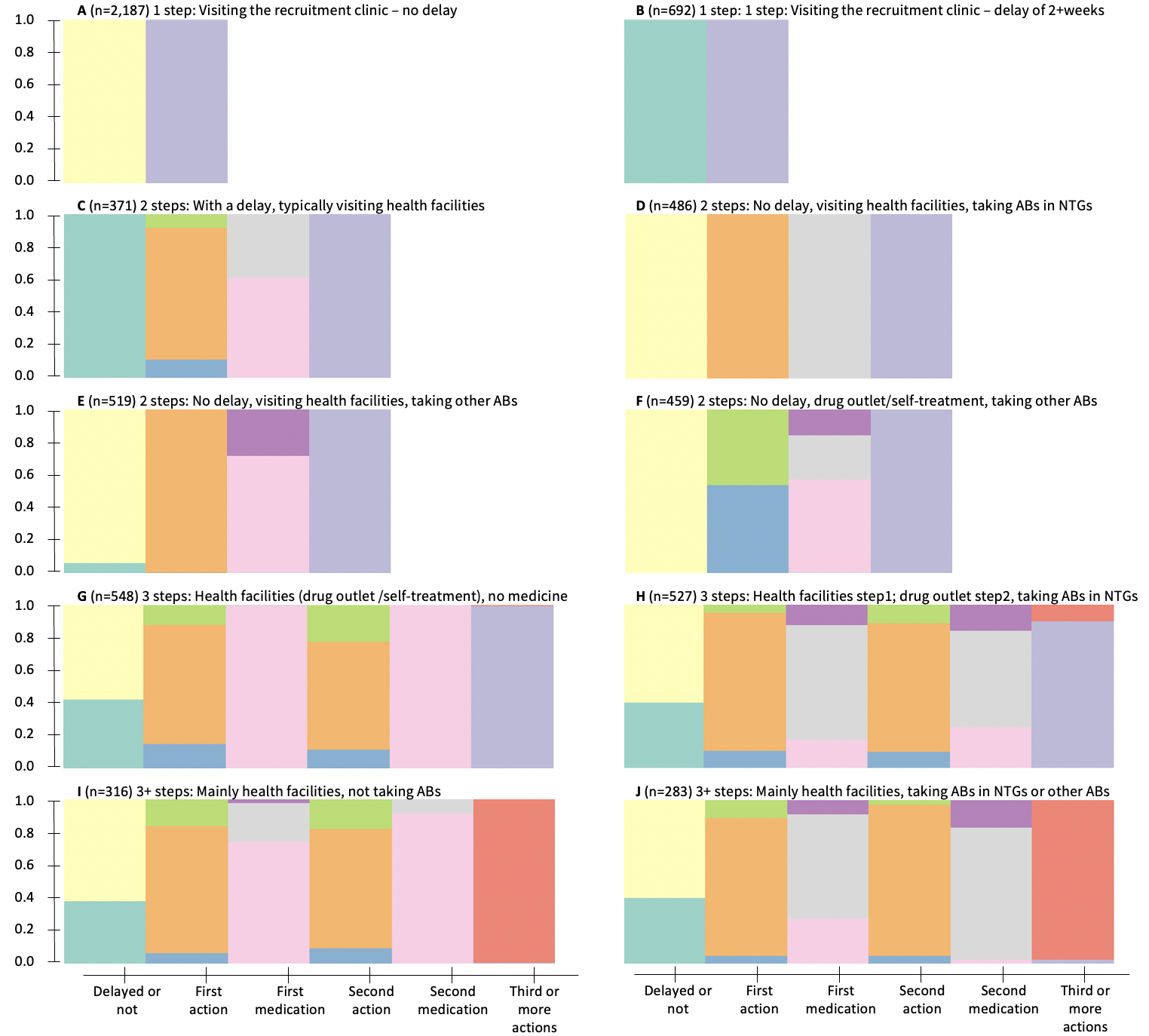


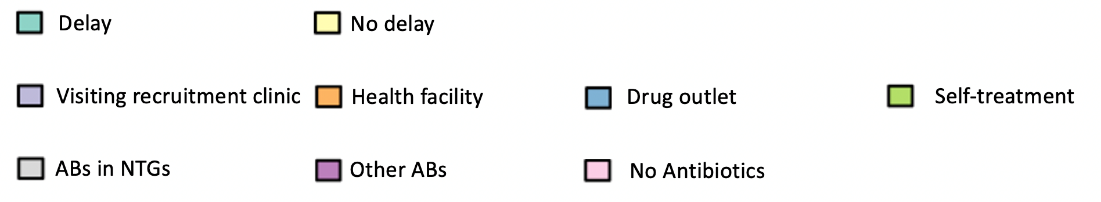


Notes: The x axis represents pathway steps and characteristics, and the y axis shows the proportion of individuals in the cluster with that pathway characteristic. For example, all patients in cluster D had no delay, went to healthcare facilities as their first step, and got ABs from NTGs, before visiting the recruitment clinics. Subjects included in cluster F also had no delay, but for the first step over half visited pharmacies/drug shops, and some of those obtained ABs, or no ABs, before showing up at the recruitment clinic.
